# From good health to illness with post-infectious fatigue syndrome: a qualitative study of adults’ experiences of the illness trajectory

**DOI:** 10.1186/s12875-017-0614-4

**Published:** 2017-03-27

**Authors:** Eva Stormorken, Leonard A. Jason, Marit Kirkevold

**Affiliations:** 1 Department of Nursing Science, Institute of Health and Society, University of Oslo, P.O.B. 1130 Blindern, 0318 Oslo, Norway; 2 Center for Community Research, DePaul University, Chicago, IL USA

**Keywords:** Disability, Chronic fatigue syndrome, In-depth interview, Myalgic encephalomyelitis, Natural course, Patient experiences, Primary healthcare, Qualitative research

## Abstract

**Background:**

Municipal drinking water contaminated with the parasite *Giardia lamblia* in Bergen, Norway, in 2004 caused an outbreak of gastrointestinal infection in 2500 people, according to the Norwegian Prescription Database. In the aftermath a minor group subsequently developed post-infectious fatigue syndrome (PIFS). Persons in this minor group had laboratory-confirmed parasites in their stool samples, and their enteritis had been cured by one or more courses of antibiotic treatment. The study’s purpose was to explore how the affected persons experienced the illness trajectory and various PIFS disabilities.

**Methods:**

A qualitative design with in-depth interviews was used to obtain first-hand experiences of PIFS. To get an overall understanding of their perceived illness trajectory, the participants were asked to retrospectively rate their functional level at different points in time. A maximum variation sample of adults diagnosed with PIFS according to the international 1994 criteria was recruited from a cohort of persons diagnosed with PIFS at a tertiary Neurology Outpatient Clinic in Western Norway. The sample comprised 19 women and seven men (mean age 41 years, range 26–59). The interviews were fully transcribed and subjected to a qualitative content analysis.

**Results:**

All participants had been living healthy lives pre-illness. The time to develop PIFS varied. Multiple disabilities in the physical, cognitive, emotional, neurological, sleep and intolerance domains were described. Everyone more or less dropped out from studies or work, and few needed to be taken care of during the worst period. The severity of these disabilities varied among the participants and during the illness phases. Despite individual variations, an overall pattern of illness trajectory emerged. Five phases were identified: prodromal, downward, turning, upward and chronic phase. All reached a nadir followed by varying degrees of improvement in their functional ability. None regained pre-illness health or personal and professional abilities.

**Conclusions:**

The needs of persons with this condition are not met. Early diagnosis and interdisciplinary rehabilitation could be beneficial in altering the downward trajectory at an earlier stage, avoiding the most severe disability and optimising improvement. Enhanced knowledge among health professionals, tailored treatment, rest as needed, financial support and practical help would likely improve prognosis.

**Electronic supplementary material:**

The online version of this article (doi:10.1186/s12875-017-0614-4) contains supplementary material, which is available to authorized users.

## Background

In 2004, the parasite *Giardia lamblia* contaminated the municipal drinking water reservoir supplying a part of Bergen City, Norway, due to a leakage of faecal matter from a sewer pipe [1–3]. *Giardia l.* is endemic in many countries globally, but not in Norway [2]. This parasite induces enteritis, a diarrhoeal disease [3]. One of the two waterworks supplying tap water to the city, Svartediket, supplies 52.000 inhabitants. People who lived, worked or had stayed in that part of the city during the outbreak were exposed to the contaminated water. A report estimated that between five to six thousand people were infected [1]. The laboratory at Haukeland University Hospital identified the total of 1262 cases of confirmed *Giardia l.* parasites in stool samples [4]. According to the Norwegian Prescription Database more than 2500 persons with Giardia duodenalis were treated with antibiotics following the outbreak [2]. Most were women and younger persons. The only predictor for falling ill was drinking more than five glasses of contaminated water daily [2, 5]. Antibiotics cured the infection, but many affected individuals developed persistent tiredness and irritable bowel syndrome (IBS) [6, 7]. A minor group developed debilitating fatigue and was subsequently referred to the hospital’s Outpatient Neurology Clinic from August 2005 to September 2007 with suspicion of post-infectious fatigue syndrome (PIFS) [4].

The term PIFS refers to severe and prolonged fatigue following infectious triggers [8], such as viruses [9], bacteria [10] and parasites [4]. Because infectious agents are not always objectively established, the terms post-viral fatigue syndrome (PVFS) [11] and myalgic encephalomyelitis (ME) [12] have also been used for this condition. For convenience the term PIFS will be used hereafter. The most frequent onset is acute [13]. Within days or weeks of the initiating infectious trigger, which is termed the prodromal phase, the affected individuals experience a progressive decline in functional ability. A minor group has a more gradual or insidious onset, and the decline may take many months or years. In both cases, the decline in health is followed by the emergence of numerous new symptoms from different body systems. The fatigue must have resulted in a 50% reduction in functional level compared with pre-illness and have persisted for more than six months in order to define it as PIFS [13, 14]. Rapid fatigability, pain, neurological complaints and autonomic and immunological dysfunction are prominent features. Most characteristic is post-exertional malaise (PEM) manifested as symptom exacerbation followed by increased disability induced by any kind of physical, cognitive or emotional exertion. This exacerbation and the increased illness burden can last for weeks [15, 16]. Although pathologic mechanisms [12] such as dysfunctions in the immune system [17, 18] and neuroendocrine system [19], chronic inflammation [20], an increase in gene expression following exercise [21] and brain inflammation [22] are found, the aetiology remains unclear. A heritable predisposition is suggested [23]. No cure is currently available; therefore, treatment aims at alleviating symptoms and fostering the mastery of challenges in daily life to improve functioning [24–26]. A review of natural courses found that complete recovery is rare [27] - less than 10% - but that the odds for improvement of symptoms may be more favourable. The frequency of resuming work ranges from 8–52% [27] and for living on disability benefits 25–42% [28, 29]. Even individuals who claim to have recovered are unable to resume pre-illness activity levels [30]. Individuals with PIFS experience multiple functional losses and disabilities as a result of the illness. The disabilities may be permanent, chronic, episodic or fluctuating in nature. Population-based prevalence is around 0.4% [31].

The illness course and its phases are also worth exploring, because knowledge in this area may offer insights for a better approach to understanding this chronic condition and identifying the challenges in each phase, which could further the development of more appropriate phase-specific treatments. When taking into account the relapsing and remitting nature of PIFS [13], a model of discrete and hierarchical stages that presupposes succeeding and fixed stages seems inappropriate. A previously described model for understanding the phases in clinical practice is the Fennell Phase Inventory (FPI) [32]. The FPI comprises four phases termed Crisis, Stabilisation, Resolution and Integration, and it focuses on the ways that affected individuals cope during each phase. It is progressive, but flexible, attempting to capture the condition over time, allowing for overlap between phases, regression to an earlier phase or being in more than one phase at a given time [33]. Previous quantitative research has explored the FPI phases and found support for its use [33–36]. However, the personal experience of disability that accompanies the transition from good health to contracting an infection and subsequently living with PIFS has not been explored empirically. Thus, a model to understand the illness phases and fluctuations in functional level is needed. General practitioners (GPs) may feel uncomfortable in making the diagnosis or defining the condition [37], and health professionals have a limited understanding of it [38]. Because appropriate advice and support from health care professionals may vary during the illness trajectory, there is a need to understand how the disabilities are experienced in different phases and how the severity of different disabilities is experienced by the persons affected. The purpose of our study was to increase our understanding of how the participants experienced the evolvement of their illness trajectory from the onset of the *Giardia l*. enteritis and over the subsequent four years when looking back on their experiences.

## Method

### Design

This study had a retrospective explorative qualitative design [39, 40]. We employed in-depth qualitative interviews to gain access to the participants’ experiences and conducted an inductive qualitative content analysis [41].

### Sample

Among severely fatigued individuals referred to the Outpatient Neurology Clinic [4] a cohort of laboratory confirmed *Giardia l.* parasites in their stool samples was diagnosed with PIFS by a neurologist according to international criteria [14]. This Giardia PIFS cohort, that is, the population, accounted for five percent of the total number of laboratory confirmed cases [4]. Four years after the outbreak in 2004, the interview sample was derived from this cohort of 58 ethnic Caucasian Norwegians [4]. A request for participation, consent form, questionnaires regarding demographic and socioeconomic variables, number of signs and symptoms [42] [see Additional file 1], and Bell’s Disability Scale to retrospectively record functional level at different points in time [43] [see Additional file 2], were administered from the outpatient clinic to the Giardia cohort in January 2008. Forty-four patients (76%) answered the questionnaires. Based on differences in gender, age, education level, income, work/study status, symptom burden, and functional level, we selected a maximum variation sample of 26 persons among those 44 who had returned the questionnaires [44]. We then contacted 19 women and seven men for an in-depth qualitative interview. None of the invited participants who consented to being interviewed withdrew. Mean age for the sample was 40.9 (range 26–59), whereas mean age for women was 41.4 years (range 26-59) and for men 39.5 years (range 26–58). Twelve were single, nine married, two cohabiting and three divorced. Education level varied: two had completed their education up through junior high school, four through senior high school, six through their college/university undergraduate degree and 14 through their college/university graduate degrees. Prior to the *Giardia duodenalis* infection, all were engaged full time in work or studies. The number of symptoms from various body systems [42] ranged from 14 to 70 (median 36). In the questionnaire the participants were asked to describe the household income according to five categories: Four perceived themselves to have very low income, eight low, nine average, six high and one very high three months before the interview. Some reported full symptomatic recoveries from their *Giardia d.* infection; however, 19 (14 women and 4 men) continued to have symptoms of irritable bowel syndrome (IBS). Sixteen (12 women and 4 men, 61.5%) reported having received a physician-confirmed diagnosis of post-infectious IBS. None had IBS prior to the Giardia enteritis. In addition to in-depth interviews, the participants were asked to retrospectively rate their ability to function on Bell’s Disability Scale [43] [see Additional file 2], a clinical measure recommended for use in primary care [45] and used in other studies [46, 47] (see the Result section). The purpose of this was to get an overall understanding of their functional abilities during the course of their illness.

### Procedure

To reduce the effort of participation, we conducted the in-depth interviews prior to a scheduled follow-up appointment at the Outpatient Neurology Clinic, four years after the Giardia outbreak. All interviews were audiotaped and lasted from one to two hours (mean 1.5 h). The aim of the interview was to explore the participants’ personal experiences. An interview guide [see Additional file 3] derived from previous research and clinical experience was used to ensure that all participants conveyed their experiences in the same areas. The opening question was “Please tell me about your life, from being healthy in the spring of 2004 until today”. Follow-up questions and prompts were used to explore their experiences in more depth, for example: “Please describe how the condition has influenced your work, studies, leisure activities or everyday life during the trajectory”. To further explore the experiences, the following prompts were used: “Can you elaborate on specific incidents or provide examples of how your functional level affected your everyday life”?

### Data analysis

As a first step, the transcripts were read multiple times to obtain a preliminary understanding of the participants’ experiences and the context [48]. During this initial exploration of the interview data we observed that the participants described a fluctuating illness trajectory. In addition we noted a pattern of fluctuations in various functional disabilities. We then used NVivo 10 software [49] to extract material pertaining to the research question, including sentences and text passages [50]. As the second step we used a manual inductive analytic approach to the extracted text to avoid preconceived categories [51, 52]. Upon reading the extracted text, notes and open codes were written down in the margins [50]. In the first coding cycle [53], meaning units emerging freely from the text were given descriptive code labels (Table 1).

**Table 1:** Examples of meaning units, condensed meaning units and codes

Meaning unit	Condensed meaning unit	Code
It’s a bit like a prolonged period of lethargy, which one has after flu… it seemingly wasn’t a disease.	Condition perceived as a common flu	Tiredness
I collapsed very quickly. I think my working hours were reduced to one hour every 14 days.	Suddenly becoming severely fatigued	Disability onset
[My] reaction to [exertions] is excessive.	Exercise provokes symptom flare-up	Increased disability after exercise

During the second coding cycle [50], we sorted coded text into mutual exclusive categories and sub-categories (Table 2).

**Table 2:** An example of category, sub-category and descriptive codes

Category	Disability	
Sub-category	Cognitive impairments	
Codes	• A brain out of order • Brain filled with cotton • Memory loss • Short-term memory • Recall difficulties • Reading difficulties • Reduced intellectual capacity • Concentration difficulties • Inability/reduced ability to think	• Beyond time • Disorientation • Word-finding difficulties • Difficulties in constructing lines of thoughts • Fluctuating cognitive ability/stamina • Easy cognitive fatigability • Lack of cognitive stamina

We revised the categories and sub-categories several times to ensure that the illness trajectory and various disabilities reflected the participants’ experiences [50], and ended up with five different phases. The prodromal phase was described as the phase from falling ill with *Giardia l*. infection until developing symptoms characteristic of PIFS, whereas the subsequent four phases described the experienced trajectory of PIFS. An example of functional ability associated with each of the five emergent illness phases is provided (Table 3).

**Table 3:** Example of functional ability and the emerged illness trajectory

Category	Illness trajectory				
Sub-categories	Trajectory phases				
	Prodromal	Downward	Turning	Upward	Chronic
Category					
Functional ability	“Only fourteen days after I got Giardia… I was knocked out”	“If I walked a short distance, I needed several stops”	“I gradually got worse until… bedbound”	“I have gradually started to become better”	“Now it is fifty-fifty for me, working 50%… 50% welfare benefits”

Our data-driven analysis revealed overall iterative patterns of illness trajectory and disabilities.

### Trustworthiness

Guba and Lincoln’s five criteria for assessing the quality of naturalistic research [39, 54], credibility, dependability, conformability, transferability and authenticity were used to ensure trustworthiness [55]. To ensure c*redibility* [39], we selected a maximum variation sample to capture the range and variations of first-hand illness experiences [44, 56, 57]. The interviews had open-ended questions, allowing the participants to speak freely, using their own logic [44]. Saturation was reached as no new concepts emerged regarding the illness trajectory and disabilities after reading and interpreting several interviews. *Dependability* [39] was ensured by conducting the interviews in a conversation-like manner to establish rapport, and unclear statements were asked to be clarified. The interviewer strove to listen attentively with an open mind [58]. Field notes were recorded immediately after each interview [40]. Content analysis was used to reduce the large amounts of interview texts [51], whereas NVivo was used for a manual and inductive analysis of the transcribed interviews. Paragraphs were coded in NVivo for a subsequent manual analysis of extracted material. Transcripts were used as the standard to check the data analysis and the interpretations against [39]. A reflexive journal was used throughout the study [59, 60] to reduce researcher bias [61]. The first author conducted all the interviews, transcribed the audio-recordings and checked for consistency between audio recordings and transcripts. This author is a registered nurse with long professional experience with this patient group. Professional and personal experience with the condition constituted the first author’s preconception [62], which was reflected on before enrolment [63] and throughout the study. The second author is an experienced researcher in the field, and the third author’s preconception was influenced by previous research in other disease populations, including some experience with PIFS. *Confirmability* [39] was ensured by authors one and three reading the interviews, extracting data and coding separately [55]. Disagreements on the interpretation of meaning units, code labels and categories were discussed until agreement was reached. The second author read the final result section and agreed to the findings. We assigned quotes from different participants to sub-categories and used triangulation of investigators, and methods, using in-depth interview and Bells’ Disability Scale. Each method corroborated the other regarding the phased trajectory. *Transferability* [39] was sought by providing detailed descriptions of all aspects of the study, helping readers to judge whether the findings would be applicable in other contexts. To ensure *authenticity* [54], the findings reflect multiple realities and differences in functional ability at each phase of the trajectory.

### Ethical consideration

Request letters were sent to the Giardia PIFS cohort of 58 persons. Due to project limitations, not all could be included. Thus, the selection procedure was explained in the letter [64]. Information that could compromise identity was removed from the data. Because the sample was local, small and specific we could not reveal the professional status and therefore had to use a system of aggregate professional titles [65]. Interviewing vulnerable participants may provoke distress, and the interviewer verbally obtained renewed consent when emotional reactions such as crying spells occurred. Measures were taken to minimise any inconvenience and reduce harm. The interview room was quiet and dimly lit. When increased fatigue was observed, participants were asked if they wanted to stop, but all declined. Interviews were terminated when necessary. The principles of the Declaration of Helsinki were followed throughout the study [66].

## Results

**Fig. 1:**
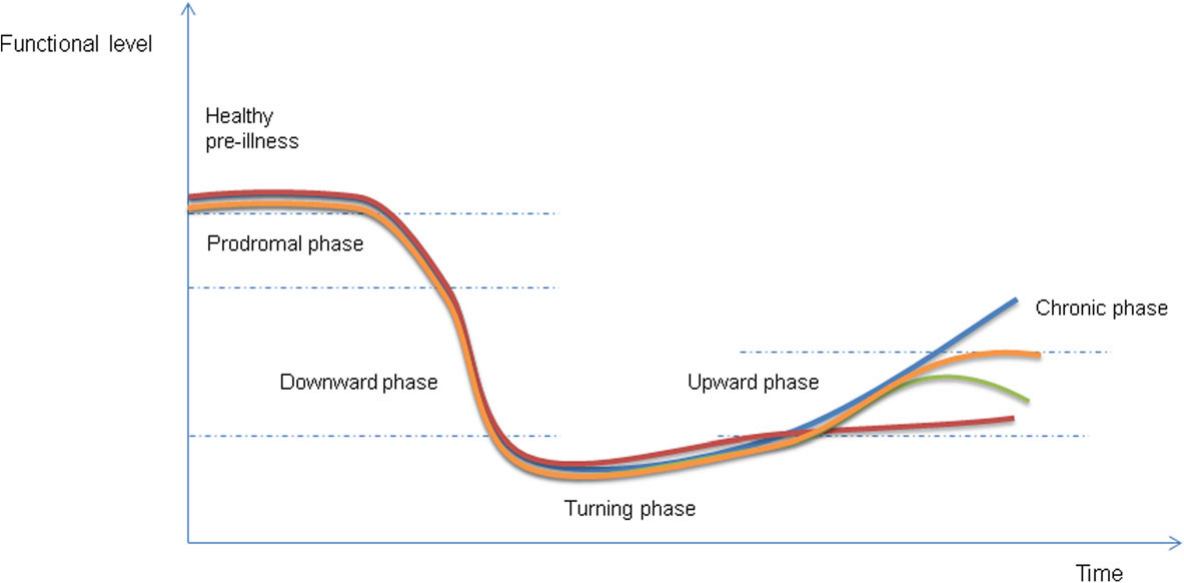
Illness trajectory phases as experienced by the participants

The participants’ accounts revealed an illness trajectory comprising five phases over the four-year period: 1) the prodromal phase following the enteritis, 2) the downward phase - the development of PIFS and a decline in functional abilities, 3) the turning phase - the most severe disabilities, a phase of illness turning, 4) the upward phase - improvement in various degrees, and 5) the chronic phase - stabilization for some participants, further improvement for others and an new decline for a few Fig. 1.

The transition between the different illness phases was imperceptible, but for clarity, the illness trajectory is divided into five overlapping phases. How long the individual participant experienced being in each phase varied, but cannot be specified in detail due to the retrospective design of this study.

### The five phases of the illness trajectory

#### Phase 1: experiences of the prodromal phase and the transition into PIFS

Before the infection, they lived ordinary lives, worked or studied full time, travelled, enjoyed cultural life and were physically active, a life comparable with healthy people’s lives: “*Before I got ill I worked 100%… was engaged in leisure time activities twice a week… always something going on… was very active, exercised*” (P7). At the onset of the infection, the participants perceived it as a “normal” stomach disease expected to pass after a few days. However, the symptoms persisted from months to a year and included diarrhoea, nausea, stomach-aches and cramps, flatulence, muscle and joint pain, headaches and food intolerances:

[P]ain in the joints, headache, muscles and stomach…whole body is aching. A terrible foul-smelling stool… bloating… farting and burping all the time… 24 hours for one year (P10); I couldn’t tolerate eating anything. The kilos vanished (P7).

The largest reported weight loss was 27 k. The participants experienced a cyclic pattern and related their tiredness to the infection and running to the toilet:

You didn’t have it day in and day out. These parasites lay eggs inside us, so they have this cycle… was healthy for five days and thought it had passed, but after five, four, three days, another wave [of diarrhoea and stomach cramps] occurred… became increasingly tired (P2).

It took a long time before they were diagnosed with *Giardia d.* and treated, and many needed more than one course of antibiotics and had to have their stool re-examined:

[It took a] long [time] before [blood and stool] samples were taken… long time before I got medication (P1); I had Giardia in 2004, September-October, and I had three courses [of antibiotics] and completed the treatment in January 2005 (P13).

Although the *Giardia d.* infection was cured, the affected individuals did not fully recover as expected: “*The parasite disappeared from the body, gone, but the other symptoms didn’t*” (P25). A few described a rapid development of severe fatigue over a couple of weeks, some more gradual over months, whereas others described a slow decline in functional ability over years. The tiredness developed almost imperceptibly into profound fatigue:

[O]nly 14 days after I got the Giardia [October 2004]… [I was] knocked down almost immediately (P15); [It] just happened insidiously. In June 2006 I threw in the towel (P18); [It] started as energy [failure]… worst period in February 2007 (P17).

The participants experienced a worsening of fatigue when gastrointestinal (GI) symptoms increased and vice versa, suggesting the whole body system was involved in a vicious circle:

PIFS and [giardiasis] stomach, the one amplifies the other. If I feel ill, the stomach upset gets worse. If I overexert myself, then the whole system gets [worse]. It’s a little bit difficult to distinguish Giardia [from PIFS]… there is a correlation… it becomes a vicious circle (P1).

#### Phase 2: experiences of the downward phase of PIFS

The participants described the downward phase as a continuous decline in disability levels in all areas. Physical, cognitive, emotional, social and vocational exertions provoked symptom flare-ups. They experienced a dramatic change in health and had never experienced anything like it before:

It’s dramatic to go from being healthy to being like this (P23); Never, ever experienced anything like this (P18).

They felt their bodies were like an engine slowing down and failing: “*I got worn out faster, like an engine that runs slower and slower and then it stops*” (P26). There was a steady increase in new symptoms from several body systems and increasing symptom intensity:

At that time I had steadily growing symptoms (P23); [I] started to get pain everywhere (P10); I've lost my potency (P6).

Their physical functional ability and stamina severely declined. A few became bedridden or housebound. For others, it was difficult to walk short distances, even with breaks. Physical capacity was poor, and restitution time prolonged:

I gradually got worse until I just collapsed, bedridden. In January 2006 I had the final collapse… completely finished, exhausted, sick (P23); If I walked a short distance, I needed several stops (P16); [I had] very poor capacity (P26).

They also experienced decreasing cognitive ability resulting in concentration problems, difficulties with word-finding, disrupted trains of thought, reduced short-term memory and memory loss, and remembering names or faces. Their brains were perceived as “out of order”, making cognitive tasks difficult to perform:

[I] became disoriented and unable to collect my thoughts and concentrate [2005]… a layer is covering the head and the body and you notice that the brain actually doesn’t work as it used to… We’ve lived in our bodies for many years, so we know how things should be (P23).

The decreasing functional ability and increasing symptom burden, the insecurity and guilty conscience were very energy-draining and added to their burden:

Mom is sick, Mom is sick and she is sick all the time. It affects you emotionally… no desire to be a bad mother… I thought I was going to die, perish at a time when I was very ill (P10).

Some were frightened or thought they might die; some were concerned that they had not done what was best for them:

When you don’t receive information, when things are wrong, you become afraid: ‘Am I making mistakes? Am I taking a wrong step? Should I do this? Should I do that? (P11).

The participants started to experience intolerances to alcohol and foods as well as sensory and neurological symptoms:

[S]ymptoms appeared… didn’t tolerate noise, light, no sensory input at all (P9); There is a great deal of foods I can’t tolerate (P13); I started to get pain in my eyes (P10); My body started to drag somewhat to the side (P1).

They experienced that their bodies were hard to control, and pushed themselves beyond their bodies’ capacity, both in personal and professional life. This led to further decline in functional ability:

I’m unable to control [my body] (P1); I was very good at pushing myself. I can see that in retrospect (P9); I worked first 100%, then 50%, then, I crashed against the wall, quite simply (P4).

During the downward phase, the participants described a clear pattern of fluctuating and declining work or study capacity, and many dropped out temporarily or permanently: *“I came to a complete halt… didn’t function any more in April 2006*” (P26). After realising that they were no longer able to work or study full time, they had to take sick leave. Following rest and reduced work/study load, many participants felt they were less disabled and fit enough to resume prior engagements, only to realise they had overestimated themselves again, and even got worse. This was a time of trial and error to find out what their bodies could tolerate without symptom flare-ups and increased disability:

To see the GP to be granted a longer sick leave was so emotionally demanding that I couldn’t get myself to do it… pushed me back to work 100% from January until October. In October I was granted sick leave again, worked 80 and 20% sick leave… after a while I realised that I didn’t have the capacity to do that… reduced to 50% work and 50% sick leave, then 40% work and 60% sick leave. If I push myself a little more than I can tolerate, I collapse. Late summer [2007] I worked 40%, in August I had 100% sick leave… I haven’t been working since February 2008 (P7).

Many struggled to obtain welfare benefits and to comply with the demands of the social security system. This was an on-going issue and an additional energy-draining process:

I submitted the application for rehabilitation benefits. Finally, I received a decision after a good deal of fuss. The payments were supposed to happen every month, but they didn’t. Yes, it was a full-blown financial crisis (P1).

Some described a period of hypersomnia; that is, they slept for abnormally long periods or were more or less in a state “beyond time” for hours or through night and day. They could suddenly fall asleep without noticing what was going on around them. This hypersomnia period could last for weeks or several months:

I fell asleep on duty and woke up several hours later (P18); I could sleep with 30 people around me (P19); I slept for five months (P26).

Social and cultural participation became increasingly more difficult due to profound fatigue, lack of energy and stamina, making individuals unable to make contact or maintain relationships: “*I slept for 12–14 h…* s*ocial life… didn’t exist*” (P4).

#### Phase 3: experiences of the turning phase of PIFS – the worst phase

The downward trajectory eased and reached a plateau. The disability level, varying among the participants, was at its nadir during the turning phase. Most of the participants were still able to take care of personal needs or household chores at this time, but not all:

My spouse had to wash my hair, give me a bath, dry me off and put me to bed (P8); I stayed a long time with Mom and Dad… got my meals served to me (P16).

One of the most disabled participants described spending a year almost bedridden or housebound and was monitored for a long time and taken care of by family members:

I spent all of 2005 in bed. I couldn’t really take care of myself… stayed with my sister so I could survive. At your worst you need help with everything… just the opportunity to exist, because you’re unable to take care of yourself. My mother sat by my side and monitored me (P11).

Other severely affected participants were able to sit upright for some hours: *“[U]sually up every single day, a few hours*” (P17) but needed to rest a lot of the time:

Most of the time I was lying on the couch (P9); If I went up a flight of stairs, the muscles stopped working (P23); [E]mptying the dishwasher could take an hour… breaks in between… I dreaded to start something… such as making dinner, only got halfway through dinner, then I had to lie down and rest (P9); I fainted in the shower, without warning (P4).

The participants experienced severely impaired cognitive function during the worst period, but to varying degrees. They experienced little or no intellectual capacity and struggled with severe memory loss and concentration difficulties and even an inability to think at all. In the less severely ill, cognitive abilities were less impaired, and they were able to function at home but unable to work or study:

[I] just sat in a chair… stared into space (P26); If I received a message, nothing happened inside my brain, couldn’t even think. I don’t have any recollection of 2005 (P11); I couldn’t read Donald Duck (P16); [M]y head… nothing in there but a heap of cotton. It’s empty (P22).

At the nadir, energy levels seemed too low to produce psychological worries in some participants, as they experienced profound fatigue and a state of mind “beyond time.” Hours could pass completely unnoticed:

Time’s passing. If it is one hour or seven hours… don’t notice (P15); I felt it was like a coma… the concept of time disappeared completely (P19).

The sensory symptoms and symptom intensity were described as being at their worst. The intolerances to foods, light, noise and alcohol had increased to such a degree that participants had to stop drinking alcohol and avoid or minimise factors that provoked the intolerances:

From the time I was affected with Giardia, I think I almost haven’t tasted alcohol. If I had a beer, I became exhausted and fatigued. So I stopped completely (P16); [I] couldn’t even tolerate sound or music (P26); I put on my sunglasses which really are covering and shutting out the light, and I am putting my cap on if I’m going to leave the house (P21).

Neurological symptoms were also described to be at their worst and comprised balancing difficulties, inability to walk a straight line, veering to one side, tingling and paraesthesia, vision problems and pain in the eyes, joints and muscles:

I staggered in the bathroom, didn’t have balance… leaned myself towards the wall… unable to walk a straight line. [A]lmost double vision (P23); Unsteady gait… veered a little to one side… the vision was weird, in relation to the midline (P1). [P]ain in the joints… pain everywhere, excruciating pain, migrating pain (P10).

The students had dropped out, and none were able to work full time. Those who were able to work part time needed recurrent periods of sick leave to rest in order to manage working at all. Sickness absence lasted from weeks to several months or years:

From the autumn of 2005 until spring of 2006 I did not study [at all] (P16); I never actually got better… just a little compared with the worst period… did not even think about [resuming my studies] (P23); February 2007 I took sick leave from work for three months (P17).

The profound fatigue, high symptom burden, easy fatigability, lack of energy and stamina and PEM following minimal exertions made it difficult to have a social life. The participants needed to withdraw from or limit cultural activities and social interaction:

The fatigue has an all-encompassing consequence for absolutely everything. I just disappeared from everyone (P23); I’ve been at home half a year… haven’t seen anyone… very little contact… not even talks on the telephone (P8).

The severity of their illness and the profound disabilities also had a negative impact on the ability to fulfil the role of mother/father, partner or colleague:

You don’t feel like a sexy lady when you just burp and fart all the time, have pain, and you just want not to be touched. I had guilty conscience towards my partner because I couldn’t attend anything, not be social or having a sexual relationship (P10); Your partner gets a double duty (P11); ‘Can you go skiing with us now, daddy?’ But I’m unable to do it (P15); I don’t suffice as a colleague at work (P24).

#### Phase 4: experiences of the upward phase of PIFS

Several experiences indicated that the improvement phase had started, such as fewer symptoms, less need for rest, increased functional ability and more energy. The overall improvement in functional ability during the upward phase was described as slow, and relapses could still occur. Most participants were able to resume some household chores or go for a walk, because they could sustain slightly more activity:

[I’ve] gradually started to become better. It’s a long time since I have had any crashes (P1); [C]ould gradually sustain slightly more physical activity (P9).

The cognitive disability had lessened somewhat, but to varying degrees. The ability to concentrate, read, converse and communicate with others improved gradually. Participants who had been severely ill and “beyond time” at their worst now experienced more energy, allowing emotions to re-occur. Boredom was seen as a sign of improvement:

I feel that my brain functions a lot better [now]… suddenly I became aware of it… have enough energy to read what I'm interested in, in the newspaper (P13); I’ve had concentration problems… have improved somewhat (P9); I felt for the first time since I became ill… boredom… a sign of improvement (P19).

Most participants were gradually able to endure slightly more sensory stimuli, and intolerances to foods and alcohol had lessened, but not for everyone. A few continued to be very sensitive to light and noise and used blackout curtains and ear protection gear:

I feel that I gradually can tolerate more [alcohol] (P16); [I could] slightly tolerate more sensory input… listen to the radio again… watch some television… half an hour in the evening (P9); Spicy food I can’t eat if I’m drained of energy, but on a very good day (P20).

Most participants were now able to slightly increase participation in social and cultural events and to reconnect with friends:

I feel I can resume a form of social contact that I’ve put on ice. I now feel that I could book tickets to the music festival… because I’m starting to get better (P13); [A] little contact with friends by email and possibly telephone (P23); Just that I can talk with people, discuss… keep up, that gives me a lot now (P16).

Some participants experienced improvement in functional ability:

I had full-time sick leave… probably a year passed before I resumed working 20%… for quite a long time, then increased to 40% (P1); [I] feel that my health has improved. I envision that I might be able to start working again (P13).

#### Phase 5: experiences of the chronic phase of PIFS

Following a period of more or less improvement in functional ability, most participants experienced reaching a more stable situation, suggesting that a chronic phase had been reached. Whereas a few hardly improved from their worst period of disability during the turning phase and continued to be severely disabled into the chronic phase, others experienced improvement in various degrees. A new decline after some improvement also occurred.

After four years, six women and four men (38%), were able to work 20–50% or to study part time and were only partly dependent on welfare benefits. The majority, 13 women and 3 men (62%), were unable to work or study and therefore fully dependent on welfare benefits:

Now it’s fifty-fifty for me when I’m working 50%… 50% welfare benefits (P26); I’m much better… at school six hours every day, max (P16).

Participants experienced that some symptoms had abated, whilst others remained the same. Many still suffered from irritable bowel complaints:

The recent weeks have been the best in a long time… Now I sleep 10, 11 hours instead of 14 (P4); The symptom intensity has abated… but I [still] have many symptoms (P1); Stomach pain, diarrhoea and sweating all day… my body trembles, headache and my stomach growls, flatulence (P20).

The participants continued struggling to find their bodies’ capacity limits but sometimes pushed themselves too hard, provoking short-time relapses. They experienced the fluctuating nature of the functional ability in daily life as challenging. When the functional and energy levels improved, there was still a risk of pushing oneself too much:

[I] could improve for a while, and then maybe I pushed too much, and then there were other things that certainly provoked setbacks and I could get worse again (P9).

Most participants also experienced that their cognitive function had improved. They described that the physical and cognitive functions were interrelated and varied according to overall functional level:

Today I have Alzheimer’s (P20); Still I have no memory. What happened last week, it’s gone… If I don’t have papers with me, then I remember nothing (P10); The cognitive also has become very much better (P17); I’ve resumed studying… a bachelor’s program (P14); I improved physically before I improved cognitively (P19).

With increased energy levels, most participants were able to see friends and take more part in social and cultural activities:

Now, when I’ve got some energy, I’ll make contact [with friends] again (P11); I've been abroad… weekend trips with friends two, three days (PT).

One of the participants did not experience much improvement and continued to be severely affected in the chronic phase:

I’ve been homebound, lying down most of the time. The last three years have passed as one… doing absolutely nothing. [E]ven if I use blackout curtains, I need to wear eye protection. A flash of light is too much…I get worse from any concentration… watching TV or reading… starting to cold sweat and feeling dizzy (P23).

None of the participants achieved working/studying capacity beyond 50% of their pre-illness functional level. A few experienced a new decline after a period of improvement. They either had to keep working to provide for their family because the social security system did not grant them enough support, or they continued working in an attempt to regain normalcy, resulting in dropout from work:

[T]oday I’m more ill than I was when I started getting back to work (P5); I’m on disability benefits (P20); I’ve become weaker and weaker… much more fatigued, walk like a zombie at home (P10).

Nor did anyone experience regaining their pre-illness functional abilities. Fatigue, lack of energy and poor stamina made some feel old before their time: “*I feel like an octogenarian*” (P10). Some worried whether they would ever work again: “*Will I get back to work? Is this how my life will be for the rest of my life?* (P22). Regardless of their functional levels, the participants experienced an overall positive view of their future and strongly wished to regain their health. The future was perceived to be uncertain, as they were aware of the risk of overdoing and relapses:

[It is an] uncertain world, [I] live from day to day (P10); I hope I’ll have steady progress and still get better, that this doesn’t stop now (P16).

At this point in time they had more experience with their bodies’ limitations and had altered their perspective:

Work is an important part of my identity, but I can’t spend so much energy at work that I don’t have energy enough to my family at home. I've realized it. I am conscious that I have to share the energy between my job and my family because they are equally important (P26).

### The participants’ self-rated functional levels

**Fig. 2:**
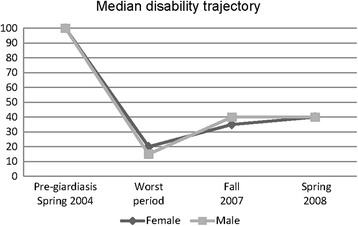
The participants’ perceived disability levels at different points in time* (*n* = 26). *Changes in ability to function during the illness trajectory were retrospectively self-rated on Bell’s Disability Scale in January 2008, before the interview. The point in time when the ability to function in everyday life reached its nadir differed among the participants occurred sometime between 2004 and 2007, thus not specified in the figures

**Fig. 3:**
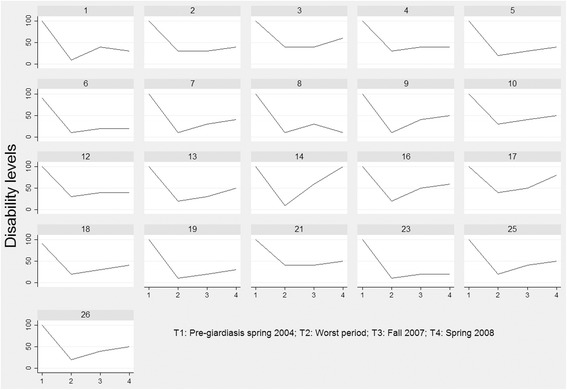
Individual illness trajectories as experienced by 21 of 26 participants. *The x-axis shows disability levels on Bell’s Disability Scale from 0–100. The y-axis displays points in time of measurement. The number on top of the individual trajectories is consistent with the patient ID number that corresponds with the participant number attached to the quotes (ID no. 1 = P1 etc.)

The participants had retrospectively rated their ability to function at some points in time on Bell’s 10-point disability grading scale [43], a scale ranging from 0 to 100 [see Additional file 2]. 100 = no symptoms with exercise; normal overall activity; able to work or do house/home work full time with no difficulty and 0 = severe symptoms on a continuous basis; bedridden constantly; unable to care for self. The median score for both genders was 100 prior to contracting the infection; this was in agreement with the background data. The nadir median score for women was 20: moderate to severe symptoms at rest; unable to perform strenuous activity; overall, activity levels are 30–50% of expected; unable to leave the house except rarely; confined to bed most of the day; unable to concentrate for more than 1 h/day. The nadir median score for men was 10: severe symptoms at rest; bedridden the majority of the time; no travel outside of the house; marked cognitive symptoms preventing concentration. The median score for both genders prior to the interviews in 2008 was 40: moderate symptoms at rest; moderate to severe symptoms with exercise activity; overall activity level 50–70% of expected; not confined to house; unable to perform strenuous duties; can perform light duty/desk work 3–4 h/day; requires rest periods. The median patterns of changes in disability seems quite similar for both genders, although the median score for men was slightly lower than for women in the fall of 2007. The downward phase comprises both the prodromal phase and the first phase of PIFS, that is, from good health in the spring of 2004 to the nadir of the illness trajectory. Our intention is not to present correlational statistics in this qualitative study, but only to present median disability scores for both genders (Fig. 2) and individual disability scores (Fig. 3) as complementary sources for visualising the perceived disability at different points in time.

The participants’ scores at different points in time indicate that they experienced individual illness trajectories, a finding that corresponded with their illness accounts. To illustrate the different trajectories among the participants, a plot of their scores is presented (Fig. 3). Five of the 26 participants with missing values were excluded. Participant 14 scored 100 at time point 4, indicating full recovery. At the same time, this participant reported 52 symptoms and was dependent on 50% welfare benefits, which is more consistent with a reduced functional level than full health.

## Discussion

The aim of this study was to explore illness trajectory and concomitant disabilities. When exploring how persons with PIFS experienced the first four years of their condition we identified five distinct progressive phases of the illness and disability trajectory: prodromal, downward, turning, upward and chronic phase (PIFS Disability Model [PIFSDM]). These phases have parallels to the four-phased Fennel Phase Inventory (FPI) of progressive psychological coping spanning the physical, psychological, social and workplace performance aspects of living [32]. However, our study contributed with new knowledge by focusing on the in-depth experiences of the illness phases, specifically the fluctuations in functional abilities, including physical, cognitive, emotional, neurological, intolerances, vocation/studying, role performance, symptom burden, and fatigue severity. From the retrospective accounts, we were able to untangle variations in the experienced severity of the disabilities over four years. Disabilities may comprise physical, intellectual, cognitive, emotional, sensory and neurological impairments, or any combination of these [67], and all of these were applicable to persons in our sample.

Whereas previous research found that fatigue and pain severity seemed to be the same across the phases of chronic fatigue syndrome (CFS) [68], our findings indicate that for persons with PIFS, the symptom severity may vary in different phases. Our participants described that fatigue increased during the downward phase and that it was at its worst during the turning phase; for some, the fatigue abated during the upward and chronic phases.

Most of the participants felt they were continuing to improve their functional abilities during the upward and chronic phases. However, a few participants perceived that their functional levels remained almost the same from the turning phase and onward. Others experienced some improvement, but then a new decline in functional abilities occurred during the chronic phase, apparently related to over-engagement in work-related roles. The chronic phase showed different forms, and as seen in CFS populations [33, 34], regression occurred. CFS and PIFS are relapsing and remitting conditions, and both FPI and PIFSDM describe regression to an earlier phase when relapse occurs, or a new decline in functional ability may take place in the chronic phase. In addition, there is overlap between the phases. The FPI is a system model where sociocultural factors play a significant role in the coping process, whereas the focus of PIFSDM is on how the participants experience their fluctuating ability to function in daily life. The FPI and our PIFSDM may serve as complementary sources to understand how the condition affects the everyday experiences of persons who live with it and to provide tools for assessments and treatments.

The participants exceeded their capacity limits, especially during the first phases. Ramsey noted during the outbreaks in the 1950s and later that affected individuals for whom rest was enforced when they fell ill had the best prognosis [69, 70]. This suggests that a faster progression to the turning and upward phases may contribute to less severe disabilities and facilitate improvement.

Our participants associated continued full-time work or studies for longer periods of time with higher levels of disability during the downward phase. Although this study cannot establish causal relationships, their experiences suggest that they might have needed help to ensure adequate rest early on to limit further deterioration. The participants described that the chronic phase could take many forms such as deterioration, no improvement and varying degrees of improvement. This is in line with previous findings [27, 70, 71].

Although the degree of severity and symptom burden differed among our participants, the interview data did not indicate that any of the background factors seemed to significantly influence the illness trajectory or types of disabilities. Our sample is consistent with the Giardia PIFS cohort study [4] regarding high education levels and high levels of disability, and is also consistent with other study samples regarding demographic characteristics such as education level, age and gender [2, 72, 73].

Independently of gender, age and educational level, the participants experienced the same trajectory of five phases. In our qualitative study it is not possible to know to what degree those or other variables impacted the severity of the disabilities in each phase or why some developed PIFS within a short time, whereas in others this development took a much longer time. That the time to develop PIFS varied, was also found in the Giardia PIFS cohort [4]. However, low income seemed to add to the burden and forced participants to push themselves to work in order to survive, resulting in an emotionally draining situation and lack of rest, followed by symptom flare-ups and increased disability that may have hampered improvement [74].

All fatigue is not the same [75, 76], and the time to reach a diagnosis may take years. Contextual factors such as lack of knowledge and trivializing the participants’ symptoms may have caused diagnostic delay that may have had a negative influence on the prognosis. The participants struggled on their own, had no knowledge of what was wrong with them and thus chose many counterproductive actions that made them worse. Early recognition of the illness experience, trajectory and various disabilities accompanying PIFS may be considered as a key factor for GPs and other health care providers who will be able to provide more appropriate advice on how to master the condition and its implications and offer symptom alleviation at an earlier stage. An early diagnosis for the persons affected with PIFS is also important because they will be able to understand what is wrong with them and early on find the best ways deal with their illness and disabilities to improve their health and ability to function, or regain pre-illness life.

The chance to regain full health after PIFS is slim, especially after being ill for more than two years [27]. Just when the illness trajectory progresses from an upward trend into a chronic phase is difficult to establish, as the transition may be imperceptible and biomarkers are lacking. A recent study found that the early-altered immune signature of both pro- and anti-inflammatory cytokines changed after three years [77], a factor that may indicate the transition to a chronic phase. Our findings of an overall phased illness trajectory and that the participants experienced a natural turning phase suggests a natural course of PIFS.

The variations in illness levels and trajectories compel us to concur with the notion that determining accurate prognosis of recovery on an individual level is not possible due to lack of biomarkers and a poorly understood aetiology [78]. In line with our findings, differences in functional ability and improvement remain several years after the triggering event [79], as do high rates of dropout from work [27, 80] and reliance on disability benefits [28, 29]. Our findings that some of the participants were able to work or study part time concur with previous reports [4, 27].

*Giardia l*. as a triggering agent has been suspected before [81], and a prodromal phase of gastroenteritis has been documented [13]. New intolerances to foods, alcohol and sensory stimuli have also been reported previously [13, 82] as have periods of hypersomnia [83] and a fluctuating pattern [13].

To our knowledge, this is the first qualitative study that explores the experienced illness trajectory of PIFS following a confirmed infection. The participants’ experiences add an important lifeworld perspective to the pathophysiological underpinnings and to quantitative studies on functional status that show that functional capacity is significantly lower than the general population or other disease comparison groups [84–86].

Persons with this condition experience serious impacts on their personal and professional life [87], but their needs are not being met [88]. Health care providers need more knowledge of the possibility for developing PIFS after infections, the characteristics of PIFS and its functional trajectory. It is important to identify the different phases when assessing the individual’s need for help and considering treatment options. Enhanced knowledge on the part of health care providers may result in earlier diagnoses, better prognoses [89], more appropriate advice on how to treat or manage this condition and avoidance of confusion between PIFS-associated fatigue and depression [90]. Practical assistance, granted absence from work or studies, financial support and other means of help to promote health need to be considered. Because PIFS patients have complex needs, timely and tailored treatments and support from an interdisciplinary team would help if these patients are to regain or improve functional abilities [91]. Early diagnosis and appropriate help may limit the disabilities following PIFS and may facilitate improvement.

### Strengths and limitations

The strengths of this study are the relatively large qualitative maximum variation sample, a confirmed infectious trigger, and that participants were not recruited from support groups that may confer biases in terms of special attitudes or views on, or a joint understanding of, the condition. However, our study has several limitations. Although we used maximum variation sampling, this is a sample recruited from the Giardia PIFS cohort at one medical centre at one point in time. Our sample might therefore not be representative for all PIFS populations. The high education and disability levels found in our study are consistent with those found in the larger Giardia PIFS cohort study. Moreover, the frequency of higher education levels, gender distributions and demographics are consistent with other research samples, but our sample may differ on other variables not examined in our study. The participants reported memory gaps; therefore, they may have forgotten experiences pertaining to the study’s purpose. A retrospective interview is open to memory bias and may reflect the participants’ conceptualisation of their experiences at the time of the interview rather than capturing their views when the experiences actually occurred. However, our findings correspond with previous research and therefore may be applicable to other PIFS cases or cases with ME. Because the condition is heterogeneous, our findings may not be applicable to all cases or subgroups, or population-based samples.

## Conclusions

Our in-depth qualitative analysis showed a phased illness trajectory and variations in disabilities among the participants. None of the participants in our qualitative study had experienced full remission, pre-illness functional level, or experienced a good outcome. Some were, however, able to work half-time because they rested between working days. These findings are consistent with the Giardia PIFS cohort study. Bell’s Disability Scale offered a visual and complementary source of information about the illness trajectory, as it corresponds with the participants’ own accounts. These accounts revealed severe multiple disabilities that made it challenging for the participants to communicate with others, fulfil roles, participate in social events, learn new things, keep working/studying and regain their health and pre-illness functional levels. These multiple disabilities severely disrupted the participants’ capacity to function in personal and professional life. Further research is warranted to explore the relevance of the PIFSDM in other populations of persons with PIFS and to explore different treatment options at different phases. Prospective, longitudinal studies should be conducted to uncover the experiences of persons as they unfold. A comparison of the functional trajectory in PIFS cases and cases with an unknown trigger mechanism would be helpful to identify any differences in trajectories.

## Additional files

Additional file 1: Signs and symptoms. (PDF 31 kb)

Additional file 2: Bell’s Disability Scale. (PDF 62 kb)

Additional file 3: The interview guide. (XML 237 kb)

## Abbreviations

CFS: Chronic fatigue syndrome
FPI: Fennel phase inventory
Giardia d.: Giardiasis duodenalis
Giardia l.: Giardia lamblia parasite
GP: General practitioner
IBS: Irritable bowel syndrome
ME: Myalgic encephalomyelitis
PEM: Post-exertional malaise
PIFS: Post-infectious fatigue syndrome
PIFSDM: Post-infectious fatigue syndrome disability model
PVFS: Post-viral fatigue syndrome
