# Supplementary material for: From good health to illness with post-infectious fatigue syndrome: a qualitative study of adults’ experiences of the illness trajectory
Source: BMC Fam Pract. 2017 Mar 27;18:49. doi: 10.1186/s12875-017-0614-4 (PMC5369194; doi:10.1186/s12875-017-0614-4)
Supplement: Supplementary file 1 — Signs and symptoms. (PDF 31 kb) [file 12875_2017_614_MOESM1_ESM.pdf]

APPENDIX 4. Signs and Symptoms

As the neurological, immune and endocrine systems are widely distributed, symptoms are numerous, multiform and of variable intensities. Many of the following symptoms are not present in everyone or at all times and, therefore, cannot be included as part of the criteria for diagnosis.

Circulatory System

____ neurally mediated hypotension (NMH)
____ postural orthostatic tachycardia syndrome
____ delayed orthostatic hypotension
____ light-headedness
____ palpitations
____ fluid retention
____ extreme palor
____ bruising

Digestive System

____ lump in throat
____ nausea
____ heart burn
____ abdominal pain
____ irritable bowel syndrome

Neuroendocrine System

____ loss of thermostatic stability–subnormal body temperature or diurnal fluctuations
____ hot flushes
____ excessive sweating or night sweats
____ feelings of feverishness
____ feelings of cold extremities
____ heat/cold intolerance
____ anorexia or abnormal appetite
____ marked weight change
____ hair loss

Musculoskeletal System

____ myalgia
____ muscle cramps, particularly in legs
____ chest pressure and pain
____ arthralgia
____ TMJ

Nervous System

____ persistent fatigue
____ lack of endurance
____ migraines or new onset headaches
____ seizure like phenomena

Sensory

____ hypersensitivity to pain
____ hyper-responsiveness to noxious stimuli
____ perceptual & dimensional distortions
____ feeling of burning or swelling
____ overload phenomena
____ loss of cognitive map
____ altered taste and/or smell

Cognitive

____ difficulties processing information
____ concentration problems
____ confusion
____ difficulties with word retrieval
____ word mix-ups
____ short-term memory difficulties
____ slowness in cognitive processes

Motor and Balance

____ muscle weakness or paralysis
____ poor balance, ataxia & tandem gait
____ clumsiness & tendency to drop things
____ difficulty in tandem gait
____ atypical numbness or tingling

Sleep Disturbances

____ sleep disturbance–hyper- or insomnia
____ non-refreshing sleep

Visual and Auditory Disturbances

____ photophobia
____ visual changes or eye pain
____ double, blurred or wavy vision
____ dry or itchy eyes
____ tinnitus–buzzing or ringing in ears
____ hyperacusis & cocktail party phenomena

Neuropsychological

____ loss of adaptability
____ worsening of symptoms with stress
____ emotional flattening or personality change
____ anxiety &/or panic attacks
____ reactive depression

Immune System

____ tender lymph nodes
____ recurrent sore throat
____ recurrent flu-like symptoms
____ new sensitivities to medications, chemicals

Reproductive System

____ dysmenorrhea
____ PMS or irregular menstrual cycles
____ loss of sexual libido or impotence

Respiratory System

____ exertional dyspnea
____ sinusitis
____ persistent cough & wheezing

Urinary System

____ urinary frequency, bladder dysfunction
