# Supplementary material for: From good health to illness with post-infectious fatigue syndrome: a qualitative study of adults’ experiences of the illness trajectory
Source: BMC Fam Pract. 2017 Mar 27;18:49. doi: 10.1186/s12875-017-0614-4 (PMC5369194; doi:10.1186/s12875-017-0614-4)
Supplement: Supplementary file 2 — Bell’s Disability Scale. (PDF 62 kb) [file 12875_2017_614_MOESM2_ESM.pdf]

# Bell's Disability Scale

| Scores | Descriptions of<br><br>functional levels | Tick the box that matches your level of functioning |
|---|---|---|
| **100** | No symptoms at rest; no symptoms with exercise; normal overall activity level; able to work full-time without difficulty. | |
| **90** | No symptoms at rest; mild symptoms with activity; normal overall activity level; able to work full-time without difficulty. | |
| **80** | Mild symptoms at rest; symptoms worsened by exertion; minimal activity restriction noted for activities requiring exertion only; able to work full-time with difficulty in jobs requiring exertion. | |
| **70** | Mild symptoms at rest; some daily activity limitation clearly noted. Overall functioning close to 90% of expected except for activities requiring exertion. Able to work full-time with difficulty. | |
| **60** | Mild to moderate symptoms at rest; daily activity limitation clearly noted. Overall functioning 70%-90%. Unable to work full-time in jobs requiring physical labour, but able to work full-time in light activity if hours flexible. | |
| **50** | Moderate symptoms at rest. Moderate to severe symptoms with exercise or activity; overall activity level reduced to 70% of expected. Unable to perform strenuous duties, but able to perform light duty or desk work 4-5 hours a day, but requires rest periods. | |
| **40** | Moderate symptoms at rest. Moderate to severe symptoms with exercise or activity; overall activity level reduced to 50%-70% of expected. Not confined to house. Unable to perform strenuous duties; able to perform light duty or desk work 3-4 hours a day, but requires rest periods. | |
| **30** | Moderate to severe symptoms at rest. Severe symptoms with any exercise; overall activity level reduced to 50% of expected. Usually confined to house. Unable to perform any strenuous tasks. Able to perform desk work 2-3 hours a day, but requires rest periods. | |
| **20** | Moderate to severe symptoms at rest. Unable to perform strenuous activity; overall activity 30%-50% of expected. Unable to leave house except rarely; confined to bed most of day; unable to concentrate for more than 1 hour a day. | |
| **10** | Severe symptoms at rest; bedridden the majority of the time. No travel outside of the house. Marked cognitive symptoms preventing concentration. | |
| **0** | Severe symptoms on a continuous basis; bedridden constantly; unable to care for self. | |

Bell DS. The measurement of disability. In: The doctor's guide to chronic fatigue syndrome. 2nd ed. Massachusetts: Perseus Books; 1995. p. 119-31.
